# Real-world effectiveness and retention rate of upadacitinib in patients with rheumatoid arthritis: results from a multicentre study

**DOI:** 10.1007/s10238-025-01578-2

**Published:** 2025-02-05

**Authors:** Caterina Baldi, Stefano Gentileschi, Francesca Li Gobbi, Massimiliano Cazzato, Andrea Delle Sedie, Carla Gaggiano, Emilio D’Ignazio, Gemma Lepri, Chiara De Lorenzo, Carlotta Nannini, Laura Niccoli, Anna Panaccione, Luca Di Cato, Andrea Di Matteo, Andrea Picchianti-Diamanti, Serena Guiducci, Bruno Frediani, Maurizio Benucci

**Affiliations:** 1 Rheumatology Unit, Department of Medicine, Surgery and Neurosciences, University Hospital of Siena, Viale Mario Bracci 16, 53100 Siena, Italy; 2 Rheumatology Unit, S. Giovanni Di Dio Firenze Hospital, Florence, Italy; 3 Rheumatology Unit, Department of Clinical and Experimental Medicine, University of Pisa, Pisa, Italy; 4 Division of Rheumatology, Department of Experimental and Clinical Medicine, University of Florence, Florence, Italy; 5 Department of Clinical and Molecular Medicine, Sapienza University of Rome, S. Andrea University Hospital, Rome, Italy; 6 Division of Rheumatology, Hospital of Prato, Prato, Italy; 7 Rheumatology Unit, Santa Maria General Hospital, Terni, Italy; 8 Leeds Institute of Rheumatic and Musculoskeletal Medicine, University of Leeds, Leeds, UK

**Keywords:** Upadacitinib, Janus kinase inhibitors, Rheumatoid arthritis, Cohort studies, Treatment outcome

## Abstract

This study evaluates upadacitinib (UPA) effectiveness and drug retention rate (DRR) in patients with rheumatoid arthritis (RA). Multicentre prospective observational study. Consecutive patients with RA receiving UPA were evaluated at 0, 3, 6, 12, 18, and 24 months of treatment. Key outcomes included UPA DRR and changes in clinical and serological measures over time. The study included 215 patients (72.6% female sex, mean age 60.1 ± 11.7 years). The DRR of UPA was 91.6% (95% CI 88.0–95.4%) at 6 months, 84.6% (95% CI 79.8–89.7%) at 12 months, 80.3% (95% CI 75.0–86.0%) at 18 months and 80% (95% CI 75.0–86.0%) at 24 months. UPA DRR was similar between monotherapy and methotrexate combination (p = 0.47), and across different treatment lines (p = 0.58). A statistically significant improvement from baseline was observed over 24 months considering erythrocyte sedimentation rate, C-reactive protein (CRP), Health Assessment Questionnaire (HAQ), Disease Activity Score (DAS)28-CRP, Physician’s (Ph) and Patient’s (Pt) Global Assessment (GA), Visual Analogue Scale (VAS) Pain, Simplified and Clinical Disease Activity Index (SDAI and CDAI) (p < 0.00 for all of them). Patients discontinuing UPA were more likely to be male (p = 0.02), with a longer disease duration (p = 0.03), higher baseline values of VAS Pain (p < 0.00), PtGA (p < 0.00), PhGA (p < 0.00), CDAI (p < 0.00), SDAI (p < 0.00) and corticosteroid dosage (p = 0.04). This study confirms UPA effectiveness in managing RA in the real-world practice, demonstrating sustained drug retention and improvements in clinical and laboratory measures over time. Also, UPA could be a valuable option for patients with multi-refractory RA and when monotherapy is preferred.

## Introduction

Rheumatoid arthritis (RA) is a chronic autoimmune disorder characterized by persistent joint inflammation which can lead to progressive joint destruction. RA is also associated with extra-articular manifestations, such as interstitial lung disease (ILD) and an increased risk of cardiovascular (CV) disease, along with other comorbidities [1, 2]. In clinical practice, most patients are managed effectively with conventional synthetic (cs-) or biological (b-) disease-modifying anti-rheumatic drugs (DMARDs), administered either as monotherapy or in combination regimens [3, 4]. Nevertheless, despite significant improvements in clinical and radiological outcomes, more than half of patients fail to achieve the therapeutic targets of disease remission or low disease activity due to non-response, or require treatment adjustments due to adverse events (AEs) [5–10].

In this context, patients who are refractory to first-line biologic (b)DMARDs targeting tumor necrosis factor (TNF) may benefit from therapies with different mechanisms of action, including Janus kinase (JAK) inhibition [11, 12]. JAKs are tyrosine kinases consisting in four isotypes: JAK1, JAK2, JAK3, and non-receptor tyrosine-protein kinase TYK2 [13]. Upon activation by extracellular ligand binding, JAKs initiate a signal transduction pathway essential for the expression of survival factors and molecules involved in leukocyte trafficking and proliferation. Upadacitinib (UPA), a second-generation JAK inhibitor with preferential selectivity for JAK1, was approved for the treatment of RA in Europe and the USA in 2019, based on the favourable efficacy and safety profile emerged from the pivotal registration trials [14–17]. The efficacy and safety of UPA have subsequently been evaluated in patients with moderately to severely active RA through five randomized controlled trials (RCTs), encompassing approximately 4400 patients. The SELECT clinical program, which includes the SELECT-BEYOND, SELECT-COMPARE, SELECT-NEXT, SELECT-MONOTHERAPY, and SELECT-EARLY phase III trials, assessed UPA 15 mg daily as monotherapy or in combination with conventional DMARDs, compared to placebo, methotrexate, or adalimumab. Additionally, two further studies, SELECT-CHOICE and SELECT-SUNRISE, evaluated UPA efficacy against abatacept in bDMARD inadequate responders and in Japanese patients, respectively [18–25].

Despite extensive data from RCTs, real-world evidence on the long-term effectiveness and safety of UPA in RA remains limited, particularly over extended observation periods. In a previous study by our research group, the effectiveness and safety of UPA were evaluated in RA patients using clinical and ultrasonographic data over 24 weeks of treatment [26]. In this report, we present findings from a multicentric cohort of RA patients treated with UPA, with an emphasis on long-term therapy retention, clinical effectiveness, factors influencing the therapeutic course, and the safety profile.

## Methods

This multicentric observational prospective study was conducted across seven rheumatology units in Italy.

### Aims of the study

The primary aim of this study was to evaluate UPA effectiveness in adult patients with RA expressed as (1) drug retention rate (DRR) of UPA over 24 months of treatment; and (2) changes in disease activity measures at 6, 12, 18 and 24 months of therapy (namely DAS28-CRP, SDAI, CDAI, ESR, CRP, HAQ, number of swollen joints, PhGA, PtGA, and VAS Pain).

The study secondary aims were the following:

1. To compare UPA DRR between subgroups of patients defined according to specific variables (sex, cardiovascular risk factors, history of neoplasia, therapeutic line, and baseline glucocorticoid or csDMARD use)
2. To explore the association of specific variables (sex, age, disease duration, cardiovascular risk factors, previous and concomitant therapies, history of neoplasia, RF/ACPA status, therapeutic line, and baseline clinimetric indexes) with the discontinuation of UPA in the first 24 months of therapy;
3. To describe UPA safety profile expressed as incidence of adverse events (AEs).

### Study population

We enrolled consecutive adult patients (aged > 18 years) who met the 2010 ACR/EULAR classification criteria for RA [27] and received UPA 15 mg/day following a primary or secondary nonresponse to at least one csDMARD.

UPA was prescribed either as monotherapy or in combination with methotrexate (MTX) or other csDMARDs, with or without concomitant glucocorticoids, based on standard clinical practice at each participating centre.

Patients with RA not eligible for UPA treatment according to the best practice recommendations were excluded from the study.

### Data collection

Data about laboratory and clinical evaluations were prospectively collected in a standardized format at time (T) 0 (start of UPA treatment) and after 3 months (T3), 6 months (T6), 12 months (T12), 18 months (T18), 24 months (T24) and at the last evaluation.

The following variables were collected at baseline*:* age, sex, diagnosis, date of onset of symptoms, date of diagnosis, rheumatoid factor (RF), anti-citrullinated protein antibodies (ACPA), body mass index (BMI), presence of comorbidities (hypertension, dyslipidaemia, positive history for cardiovascular events), use of oral contraceptives, previous and current drug therapies. We collected concomitant treatment with csDMARDs, as well as previous therapies with bDMARDs or targeted synthetic (ts)DMARDs employed.

In addition, the following variables were also collected at baseline and during follow up: erythrocyte sedimentation rate (ESR), C-reactive protein (CRP), swollen and tender joints, pain quantification on a visual-analogue scale (VAS), physician’s global assessment of disease activity (PhGA), patient’s assessment of disease activity (PtGA), health assessment questionnaire (HAQ), morning stiffness, Disease Activity Score (DAS28-CRP), Clinical Disease Activity Index (CDAI) and Simplified Disease Activity Index (SDAI) [28, 29], the presence of comorbidities (hypertension, dyslipidaemia, positive history for cardiovascular events), use of oral contraceptives, adverse events and adverse reactions to drugs including Herpes Zoster Virus (HZV) infections. Any concomitant treatment with csDMARDs or steroids.

### Statistical analysis

Statistical analysis was performed by using JASP open-source statistics package version 0.18.3. Descriptive statistics included sample sizes, mean and standard deviation or median and interquartile range (IQR). Shapiro–Wilk test was used to assess normality distribution of data. Differences between paired continuous variables were analysed by repeated measured ANOVA and Conover’s post-hoc test. Differences between independent continuous variables were analysed by T-test or Mann–Whitney U test. Associations between categorical variables were analysed using contingency tables with Chi-Square test with Yates' continuity correction. Dichotomic outcomes were predicted by logistic regression analysis. Time-to-event analysis was performed by Kaplan–Meier method, with the event being drug discontinuation. The survival curves were compared according to specific factors by Log-Rank test. The threshold for statistical significance was set to p < 0.05 and all p-values were two-sided.

## Results

We enrolled 215 patients, among whom 156 (72.6%) were females, 124 (57.7%) presented a positive RF and 142 (66.0%) tested positive for ACPA. Baseline demographic, therapeutic and clinical characteristics of our cohort are summarized in Tables 1, 2 and 3.

**Table 1:** Demographic and clinical characteristics of the cohort at the baseline (start of upadacitinib therapy)

Age (mean ± SD, years)	60.1 ± 11.7
Disease duration (mean ± SD, months)	134.0 ± 124.0
Females (%)	159 (72.6)
BMI	24.5 (± 3.5)
CRP (mean ± SD, mg/dl)	1.6 (± 2.3)
VAS pain (mean ± SD)	52.1 (± 22.0)
RF + (count and %)	124 (57.7)
ACPA + (count and %)	142 (66.1)
RF and ACPA + (count and %)	112 (52.1)
Cardiovascular risk factors (count and %)	50 (23.3)
HZV infection incidence (100PY)	1.4
DAS28-PCR (mean ± SD)	4.6 ± 1.1
SDAI (mean ± SD)	25.1 ± 11.8
*Comorbidities*	
Hypertension (count and %)	69 (32.1%),
DVT/PE history (count and %)	2 (0.9%)
History of Cancer (count and %)	6 (2.8%),
Hypercholesterolemia (count and %)	77 (36.5%)

ACPA anti-citrullinated protein antibodies; BMI body mass index; CRP C-reactive protein; DAS 28-CRP Disease Activity Score 28 joints —C-reactive protein; DVT Deep venous thrombosis, PE pulmonary embolism; HZV herpes zoster virus; PY Patient-year; RF rheumatoid factor; SD standard deviation; SDAI Simplified Disease Activity Index; VAS Visual Analogue Scale.

**Table 2:** Therapeutic characteristics of the cohort at the baseline (start of upadacitinib therapy)

Concomitant glucocorticoid use (count and %)	118 (54.9)
Concomitant glucocorticoid posology (median and IQR, mg/die of prednisone)	5.0 (7.5)
Concomitant csDMARDs use (count and %)	87 (40.5)
MTX	68 (31.6)
HCQ	8 (3.7)
LFN	5 (2.3)
SSZ	6 (2.8)
b/tsDMARD naive (count and %)	37 (17.2)
1 previous b/tsDMARD (count and %)	52 (24.2)
2 previous b-tsDMARD (count and %)	63 (29.3)
≥ 3 previous b-tsDMARD (count and %)	63 (29.3)

bDMARDs biological disease-modifying anti-rheumatic drugs; csDMARDs conventional synthetic disease-modifying antirheumatic drugs; HCQ hydroxychloroquine; IQR interquartile range; LFN leflunomide; MTX methotrexate; SSZ sulfasalazine; tsDMARDs Targeted Synthetic Disease-Modifying Antirheumatic Drugs.

**Table 3:** Breakdown of the previous immunosuppressive therapies used by the patients

*Previous TNFi use (count and %)*	142 (66.1)
Adalimumab	73 (34.0)
Etanercept	101 (47.0)
Golimumab	23 (10.7)
Certolizumab	29 (13.5)
Infliximab	19 (8.8)
*Previous other mechanisms of action (count and %)*	
Tocilizumab	49 (22.8)
Rituximab	15 (7.0)
Abatacept	35 (16.3)
*Previous JAK inhibitors*	30 (13.9)
Baricitinib	20 (9.3)
Tofacitinib	11 (5.1)
*Previous csDMARDs treatment (count and %)*	215 (100.0)
MTX	175 (81.4)
HCQ	50 (23.3)
LFN	36 (16.7)
SSZ	29 (13.5)

csDMARDs conventional synthetic disease-modifying anti-rheumatic drugs; HCQ hydroxychloroquine; JAK Janus kinases; LFN leflunomide; MTX methotrexate; SSZ sulphasalazine; TNFi TNF inhibitors.

All patients enrolled had failed at least one csDMARD. UPA was employed as monotherapy in 128 (59.5%) subjects while 87 (40.5%) patients were co-administered with a csDMARD: 68 with MTX and 19 with other csDMARDs. The median dosage of MTX at baseline was 10.0 (IQR 5.0) mg/week. Males in combination therapy accounted for 22/87 (25.9%) compared to 65/87 of females (74.71%). In our cohort, 37 (17.2%) patients were naïve to treatment with biologic agents or small molecules, whereas 178 (82.8%) patients had been previously treated with other biologic agents. Fifty-two patients received UPA as a second-line therapy while in 126 subjects UPA represented the third or more line of treatment. One hundred-eighteen (54.9%) patients were on treatment with systemic glucocorticoids at the start of UPA with a median dosage of 5.0 (IQR 7.5) mg/day of prednisone. The median follow-up duration was 24.0 (IQR 12.0) months.

### Upadacitinib drug retention rate

**Fig. 1:**
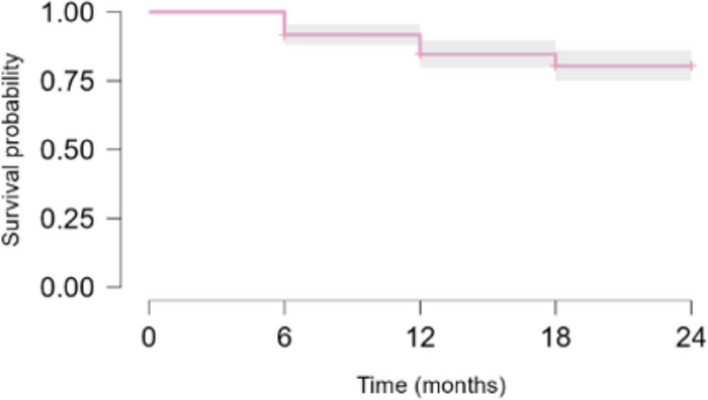
24-month survival curve of upadacitinib in patients with rheumatoid arthritis

Upadacitinib DRR was 91.6% (95% CI 88.0–95.4%) at 6 months, 84.6% (95% CI: 79.8–89.7%) at 12 months, 80.3% (95% CI 75.0–86.0%) at 18 months and 80% (95%CI 75.0–86.0%) at 24 months (Fig. 1).

Female patients showed a higher DRR than male patients (p = 0.00). There were no significant differences in UPA DRR according to the therapeutic line (p = 0.58), the previous use of bDMARDs (p = 0.98), the use of glucocorticoids (p = 0.06) or MTX (p = 0.47) at the baseline, the presence of CV risk factors (p = 0.70) or history of neoplasia (p = 0.93) at the baseline.

### Changes in disease activity indexes

**Fig. 2:**
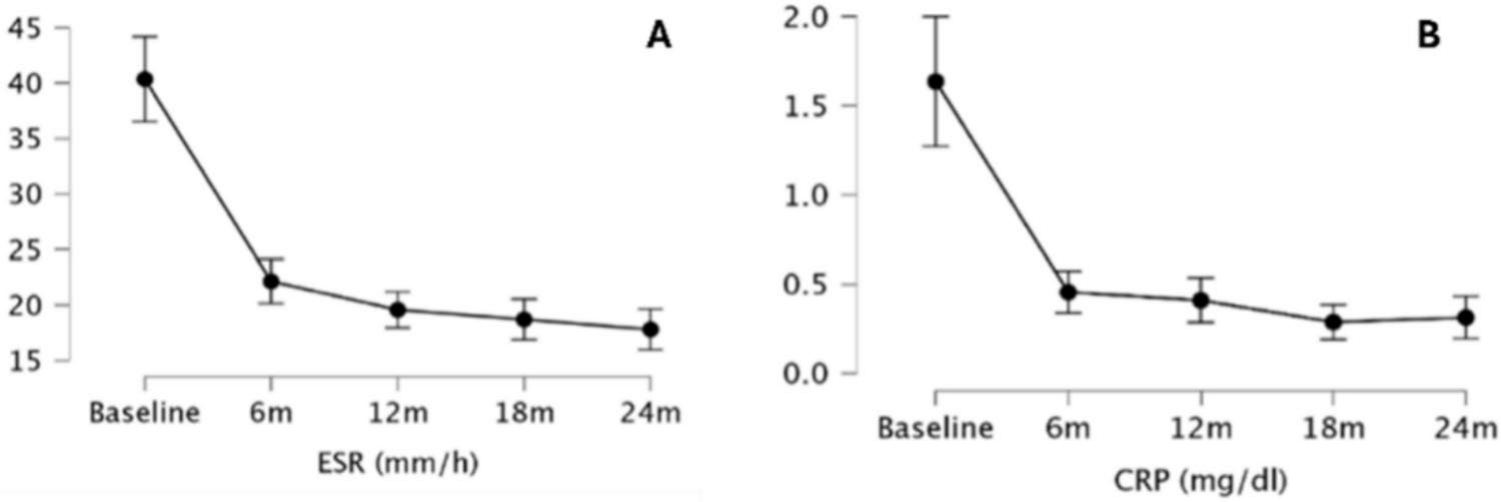
Impact of upadacitinib treatment on inflammatory markers: erythrocyte sedimentation rate (ESR) (**A**) and C-reactive protein (CRP) (**B**) HAQ, DAS28-CRP, PhGA, PtGA, VAS Pain, SDAI, CDAI, and the number of swollen joints and tender joints all showed a statistically significant improvement over 24 months of UPA therapy (p < 0.00 for all of them) (Figs. 3 and 4)

A statistically significant improvement in ESR (p < 0.00) and CRP (p < 0.00) values was observed over 24 months of UPA therapy (Fig. 2).

**Fig. 3:**
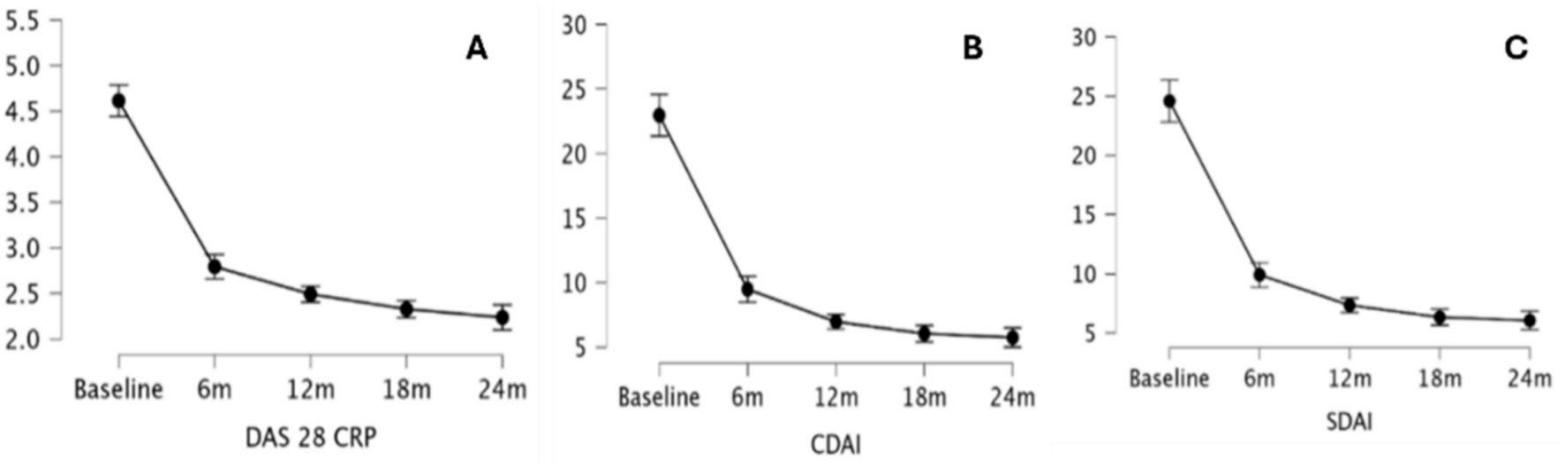
Clinimetric changes throughout the study period: disease activity score (DAS)28-C-reactive protein (CRP) (**A**), clinical disease activity index (CDAI) (**B**), and simplified disease activity index (SDAI) (**C**)

**Fig. 4:**
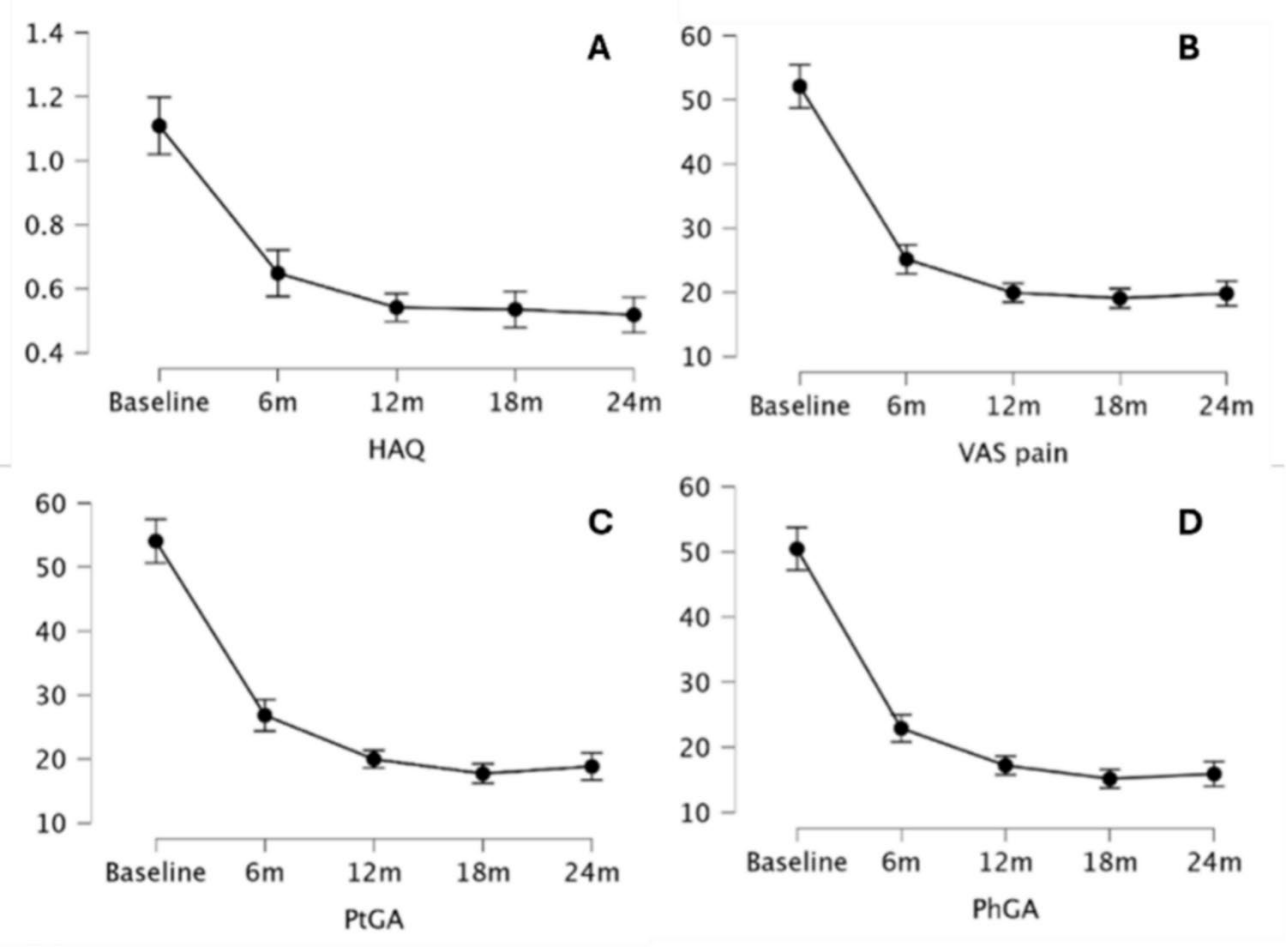
Improvement from the baseline to the last follow-up assessment on upadacitinib treatment for health assessment questionnaire (HAQ) (**A**), visual analogue scale (VAS) for pain assessment (**B**), patient global assessment (PtGA) (**C**), and physician global assessment (PhGA) (**D**)

In detail, a statistically significant improvement was observed at the Conover’s Post Hoc Comparisons between T0 and T6 and between T6 and T12 in the case of PhGA (p < 0.00 and p < 0.00, respectively), PtGA (p < 0.00 and p < 0.00, respectively), VAS Pain (p < 0.00 and p < 0.00, respectively), SDAI (p < 0.00 and p = 0.03, respectively), CDAI (p < 0.00 and p = 0.01, respectively), and the number of swollen joints (p < 0.00 and p = 0.01, respectively). In the case of ESR, CRP, HAQ, and DAS28-CRP a statistically significant improvement was observed between T0 and T6 (p < 0.00 for all the variables), but not between T6 and T12 (p > 0.05 for all comparisons). A further improvement was observed between T12 and T18 for CDAI (p = 0.03), SDAI (p = 0.01) and DAS28-CRP (p = 0.04), while none of the parameters studied improved further between T18 and T24 (Table 4).

**Table 4:** Significance levels (p-values) for clinimetric indexes comparisons between different timepoints throughout the study period

		DAS28-CRP	CDAI	SDAI	PtGA	PhGA	VAS	HAQ
Baseline	6 m	< 0.001	< 0.001	< 0.001	< 0.001	< 0.001	< 0.001	< 0.001
	12 m	< 0.001	< 0.001	< 0.001	< 0.001	< 0.001	< 0.001	< 0.001
	18 m	< 0.001	< 0.001	< 0.001	< 0.001	< 0.001	< 0.001	< 0.001
	24 m	< 0.001	< 0.001	< 0.001	< 0.001	< 0.001	< 0.001	< 0.001
6 m	12 m	0.328	0.014	0.028	< 0.001	< 0.001	< 0.001	0.118
	18 m	0.003	< 0.001	< 0.001	< 0.001	< 0.001	< 0.001	0.106
	24 m	< 0.001	< 0.001	< 0.001	< 0.001	< 0.001	0.001	0.019
12 m	18 m	0.040	0.027	0.007	0.124	0.066	0.336	0.957
	24 m	< 0.001	0.002	< 0.001	0.829	0.630	0.710	0.434
18 m	24 m	0.066	0.354	0.556	0.187	0.175	0.182	0.466

CDAI Clinical Disease Activity Index; DAS28-CRP Disease Activity Score 28 – C-reactive protein; HAQ Health Assessment Questionnaire; PhGA Physician Global Assessment; PtGA Patient Global Assessment; SDAI Simplified Disease Activity Index; T0 baseline; T6 6-month evaluation; T12 12-month evaluation; T18 18-month evaluation; T24 24-month evaluation; VAS Visual Analogue Scale.

### Analysis of treatment discontinuation

During the first 24 months of treatment, discontinuation of UPA was recorded in 40 (18.6%) patients due to primary nonresponse in 5 cases (2.3%), secondary nonresponse in 14 (6.5%), infections in 9 (4.2%), venous thromboembolism in 2 (0.9%), urticarial rash in 2 (0.9%), neoplasia in 3 (1.4%), other adverse events (Table 5) in 5 (2.3%).

**Table 5:** Breakdown of adverse events leading to UPA discontinuation

Adverse event causing UPA discontinuation	Number of events	Incidence per 100 PY
Increase of cardiovascular risk	1	0.29
Venous thromboembolism	2	0.57
Pneumonia	1	0.29
Osteomyelitis	1	0.29
Septic bursitis	1	0.29
Septic peroneal tenosynovitis	1	0.29
Urticarial rash	2	0.57
Elevated transaminases, creatine phosphokinase or lactate dehydrogenase	1	0.29
Recurrent ocular herpes	1	0.29
Optic neuritis	1	0.29
Leukopenia	2	0.57
Recurrent urinary tract infection	1	0.29
Neoplasia (lung, kidney, unspecified)	3	0.86
Genital herpes	1	0.29
Candidiasis	1	0.29

UPA upadacitinib; PY patient-year.

Patients discontinuing UPA in the first 24 months of treatment were more likely to be male (p = 0.02), with a longer disease duration at the baseline (p = 0.03) and higher values of VAS Pain (p < 0.00), PtGA (p < 0.00), PhGA (p < 0.00), CDAI (p < 0.00), SDAI (p < 0.00) and CS dosage (p = 0.04) at the baseline. Demographic and clinical factors such as age, BMI, presence of CV risk factors or neoplasia history, concomitant treatments at the baseline, fibromyalgia, ACPA/RF status, and the biological line of treatment were not associated with UPA discontinuation (p > 0.05 for all of them).

### Safety profile

Forty reports of AEs were collected, leading to treatment discontinuation in 20 cases (50.0%). The incidence of AEs was 10.9 per 100 patient-years. Five patients developed HZ during UPA treatment (incidence 1.43 per 100 patient-years), but there was no need for discontinuing therapy. No tubercular infections/reactivations were reported during the study observation. Regarding thromboembolic events, two cases of deep vein thrombosis were reported (incidence 0.59 per 100 patient-years). Three cases of neoplasia (kidney, lung, non-melanoma skin cancer) were reported (incidence 0.86 per 100 patient-years). A detailed breakdown of AEs is provided in Tables 5 and 6.

**Table 6:** Breakdown of adverse events not leading to UPA discontinuation

Adverse events	Number of events	Incidence per 100 PY
Acne	1	0.29
Elevated transaminases	3	0.86
Herpes labialis	1	0.29
Herpes Zoster	5	1.43
Elevated lactate dehydrogenase	2	0.57
Elevated cholesterol	3	0.86
Non melanoma skin cancer	1	0.29
Leukopenia	1	0.29
Anemia	2	0.57
Headache	1	0.29

PY patient-year.

## Discussion

This study offers a long-term evaluation of UPA effectiveness and safety in the treatment of RA within a real-world context, complementing data from RCTs. We observed sustained drug retention over 24 months, indirectly indicating that UPA is both efficacious and well-tolerated, contributing to continued patient adherence. This finding is further supported by the rapid and sustained improvement in disease activity markers—including disease activity indexes and laboratory parameters—and the favourable safety profile observed in our cohort.

Given the chronic nature of RA, the availability of a drug that can persist in therapy for prolonged periods of time is essential for good control of the disease, but also to allow a good quality of life for the patient. In our study, survival on treatment with UPA was 91.6% at 6 months, 84.6% at 12 months, 80.3% at 18 months and 80% at 24 months. This data appears more favourable compared to what was observed in another real-life Italian experience, in which the DRR of UPA at 24 months stood at 69.4% [30]. In the same paper, the cumulative DRR of JAK inhibitors—UPA, filgotinib (FIL), tofacitinib (TOFA), and baricitinib (BARI)—was lower in patients who were not naïve to biological/ targeted synthetic (ts)DMARDs, while in our study, the treatment line did not seem to affect patients’ persistence on treatment [31]. Concerning the concomitant use of csDMARDs, both our study and the one by Donzella and colleagues, no difference in DRR emerged between patients in monotherapy or combination treatment [30]. These findings might indicate UPA as a valuable option in patients with multi-refractory RA (i.e. difficult-to-treat RA).

Drug retention rate studies for second-line treatments in RA are highly variable and often depend on the specific drug being considered [31, 32]. However, collectively, our findings suggest that UPA may be a particularly viable option among JAK inhibitors, especially for patients who have previously failed multiple bDMARD treatments or when monotherapy is advisable or preferred by the patient. Also, they appear to challenge the traditional concept that the response rate to bDMARDs declines after multiple unsuccessful drug treatments, as observed for adalimumab in RA [33]. With this regard, it has to be emphasized that although real-world data on the first-generation JAKi is beginning to emerge, there is still a need for evidence on second-generation JAKi, which have a more selective mechanism of action [34]. A retrospective, multicentre, Italian longitudinal study on BARI included 478 patients with RA, of whom 380 (79.5%) were women. The survival rate for BARI was 94.6% at 6 months, 87.9% at 12 months, 81.7% at 24 months, and 53.4% at 48 months, which is slightly higher than the rates we observed for UPA. However, while higher therapeutic lines predicted lower DRR for BARI, UPA survival in our study was not affected by therapeutic line [35]. Similarly, in another cohort of 95 RA patients treated with BARI, previously published by our research group, a DRR of 69.3% was reported after 48 weeks of treatment. In this cohort, corticosteroid use and dosage, as well as prior bDMARD treatment, were associated with an increased risk of discontinuation [36]. A study from the Italian Study Group on Early Arthritis (GISEA) enrolled 246 patients with RA who started FIL as second or subsequent line of b/tsDMARD treatment. The survival rate of FIL was 84.5% at 6-month follow-up and 75.8% at 12-month follow-up and was comparable in both monotherapy or combination therapy, and independently from taking glucocorticoids. Patients naïve to b/tsDMARD had the lowest hazard ratio related to FIL discontinuation, while difficult-to-treat-RA the highest [37]. A study was conducted to evaluate the long-term persistence of TOFA in 23 Italian tertiary rheumatology centres, considering a treatment period of up to 48 months for all included patients. Analysis of data from 213 patients revealed that TOFA retention rate was 86.5% at month 12, 78.8% at month 24, 63.8% at month 36 and 59.9% at month 48, which appears comparable with what observed in our cohort treated with UPA. None of the factors analysed, including the number of previous treatments received, disease activity or duration, the presence of RF and/or ACPA, and the presence of comorbidities, were predictive of TOFA retention rate [38].

Our findings indicate that UPA induces a rapid and significant improvement in both objective inflammatory markers and physician- or patient-reported outcomes within the first 6 months of treatment. These improvements continue for clinical disease activity indices (CDAI, SDAI) up to 12 months, albeit at a slower pace, eventually reaching a plateau by 24 months. However, the absence of further significant improvements in objective measures, such as laboratory markers and DAS28-CRP (which incorporates CRP values), as well as in patients’ functional ability, between 6 and 12 months suggests that the majority of therapeutic gains are achieved early in the treatment course. This highlights that UPA provides a robust initial response, effectively stabilizing disease activity within the first six months, which is crucial to consider when formulating treat-to-target strategies in RA.

The UPARARemUS study, a real-life observational study, also confirmed the rapid efficacy of UPA, achieving clinical and ultrasound remission at 24-weeks in almost two thirds of enrolled patients. [39]. From our results, it can be inferred that certain clinical characteristics, such as higher baseline disease activity—as indicated by higher VAS Pain, PtGA, PhGA, CDAI, SDAI scores, and glucocorticoid usage—and longer disease duration, play a significant role in predicting the likelihood of UPA discontinuation within the first 24 months of treatment. Conversely, other factors such as age, BMI, CV risk factors, neoplasia history, fibromyalgia, and ACPA/RF status do not appear to significantly influence the likelihood of discontinuing UPA. It should be noted that, in our cohort, most cases of UPA discontinuation were attributable to AEs, which are more likely to occur in patients with higher disease activity. Additionally, patients with higher disease activity may receive additional medications (including glucocorticoids and csDMARDs), potentially increasing the rate of AEs. Finally, physicians may choose to discontinue UPA prematurely in patients with high disease activity if it is perceived as ineffective within the first months of therapy, despite the potential need for additional time to achieve full therapeutic efficacy. Disease duration appears to be a significant predictor of therapy discontinuation, potentially due to the increased likelihood of cumulative disease-related damage or side effects of therapies, or a perceived decline in treatment efficacy over time. A retrospective study by Martinez-Molina and colleagues examined reasons for drug discontinuation with the four available JAK inhibitors over 12 months of treatment [40]; the primary reasons for discontinuation among 189 treatment courses were lack of efficacy (24.3%), AEs (20.6%), and other reasons (3.7%), while 51.4% of patients continued treatment. Factors such as disease duration, disease activity, sex, seropositivity for RF or ACPA, previous biological treatments, prior JAK inhibitor use, concomitant CS use, and csDMARD use were not linked to treatment discontinuation. In detail, no patient-related factors were associated with discontinuation due to lack of efficacy, while discontinuation due to AEs was significantly higher in patients aged 65 years or older compared to those under 65 years [40]. In this regard, a recent study on RA patients treated with JAK inhibitors across three centres in Tuscany challenged the relationship between older age and a higher frequency of AEs in patients on JAK inhibitors [41]. Among 82 patients, 78.6% had at least one risk factor, including age over 65 years, obesity, smoking, hypertension, dyslipidaemia, hyperuricemia, diabetes, previous venous thromboembolism or cancer, and severe mobility impairment. A total of 28 AEs per 100 patient-years were recorded, and neither the presence nor the cumulative number of risk factors, nor age over 65, predicted their occurrence [41]. Notably, the incidence of AEs with UPA in the present cohort (10.9 events per 100 patient-years) was lower than the cumulative incidence observed with all JAK inhibitors in the previous study. This suggests that the reduced frequency of AEs associated with UPA in this cohort may not be solely explained by a lower prevalence of risk factors.

This study’s strengths include its multicentric, prospective design and extensive data collection, enhancing generalizability across diverse clinical settings. The 24-month follow-up period provided valuable insights into the sustained effectiveness of UPA, with detailed subgroup analyses offering useful information for tailoring therapy. However, as an observational study without a control group, it has limited capacity for causal inference. The high frequency of prior biologic use among participants may limit generalizability to biologic-naïve patients, and concomitant treatments, particularly glucocorticoids, introduce confounding factors that make it challenging to isolate the specific effects of UPA.

In conclusion, UPA demonstrated rapid and sustained effectiveness over 24 months in the treatment of RA in a real-world setting, with a manageable safety profile and an excellent retention rate. The findings from this study could be used to tailor therapeutic strategies and improve patient monitoring, particularly for those at risk of therapy discontinuation.

## Data Availability

All the data are provided in the main text.
